# Flight Performance, Fecundity, and Ovary Development of *Grapholita molesta* (Lepidoptera: Torticidae) at Different Ages

**DOI:** 10.3390/insects13090837

**Published:** 2022-09-14

**Authors:** Sha Su, Xiaohe Zhang, Jilong Zhang, Baojian Huang, Chengzhi Jian, Xiong Peng, Marc J. B. Vreysen, Maohua Chen

**Affiliations:** 1 Key Laboratory of Integrated Pest Management on Crops in Northwestern Loess Plateau, Ministry of Agriculture and Rural Affairs, State Key Laboratory of Crop Stress Biology for Arid Areas, Northwest A&F University, Xianyang 712100, China; 2 Joint FAO/IAEA Programme, Entomology Unit, FAO/IAEA Agriculture & Biotechnology Laboratory, International Atomic Energy Agency, A-2444 Vienna, Austria

**Keywords:** the oriental fruit moth, insect rearing, flight capacity, sterile insect technique, quality control

## Abstract

**Simple Summary:**

New environment-friendly strategies, such as the sterile insect technique (SIT), are urgently needed to manage populations of *Grapholita molesta*, a serious fruit pest. When mass-rearing the insect in a factory, assessments of various appropriate quality control parameters are essential. Flight ability, fecundity, and ovarian development are critical quality control parameters to be measured for moths being mass-produced. In this study, we found that that the flight performance, fecundity, and ovarian development of *G. molesta* moths changed with age. The fecundity of *G. molesta* at different ages was closely related to the ovarian development. *G. molesta* females began to lay eggs in large numbers from the third day after emergence. It is suggested that three-day-old males are the best option for releasing *G. molesta* for SIT. When mass-rearing and keeping the population of the moth in factory, female moths older than three days can be used for egg-laying.

**Abstract:**

*Grapholita molesta* is one of the most serious pests in fruits orchards. Flight performance of male insects and fecundity of female insects are important quality control parameters when moths are mass-reared for use in environment-friendly control strategies such as the sterile insect technique (SIT). However, information about flight performance, fecundity, and ovary development of *G. molesta* at different ages is scarce. In this study, we used a flight mill information system to measure the flight ability of female and male adults of *G. molesta* at different ages, and evaluated fecundity and ovarian development of female adults at different ages. The results demonstrated that the flight parameters (cumulative flight distance, cumulative flight time, maximum flight distance and maximum flight duration) of female and male *G. molesta* varied with age. Six-day-old female moths and three-day-old male moths were the strongest fliers, whereas the fecundity of one-day and two-day-old female moths was significantly lower than that of three to seven-day-old females. Five-day-old females had the highest fecundity. Their ovaries demonstrated mature eggs in the lateral and middle oviducts as of the third day post-emergence. It is suggested that the optimal age for moths to be released in SIT programs is three days, and moths older three days can be used for mass-rearing in a factory.

## 1. Introduction

The oriental fruit moth *Grapholita molesta* (Busck) (Lepidoptera: Tortricidae) is one of the most serious pests of stone and pome fruits [1,2]. The pest originates from eastern Asia, from where it has spread worldwide [3], causing significant annual economic losses [4,5]. This species has 3–7 generations per year depending on its location with generations overlapping [6]. *Grapholita* *molesta* can switch hosts, from peach to apple and pear in the late season [4]. The indiscriminate use of chemical insecticides has resulted in the moth developing a resistance to many of these toxic chemicals [7], i.e., methyl carbamates such as carbaryl, carbofuran, chlorpyrifos, and deltamethrin [8,9,10]. New environment-friendly strategies, such as the sterile insect technique (SIT), are urgently needed to manage populations of *G. molesta*. The application of the SIT requires the mass-rearing of the target insect in factories, sterilization of one or both of the adult sexes, and then releasing the sterile adults in the target area in numbers that are adequate to overflood the wild population [11]. Ovarian development, fecundity, and flight ability are critical quality control parameters to be measured for moths being mass-produced for SIT programs [12,13].

Ovarian development and fecundity are two important quality control parameters in the mass-rearing of insects. The ovary is the organ in the reproductive system where eggs are developing. The development of the eggs in the ovary is closely related to the fecundity of insects and is considered a potential predictor of reproductive status [14,15].

Flight ability is important for male moths to find females for mating in the field [16]. In area-wide integrated pest management (AW-IPM) programs that have an SIT component, the released sterile males need adequate flight ability to outcompete wild males and to find and mate with wild females, leading to sterility or no offspring and thus decreased wild populations [17]. Field population and laboratory strain of some lepidopteran species such as the codling moth *Cydia pomonella* (L.) (Lepidoptera: Torticidae) and *G. molesta* have an intrinsic low flight ability, so releasing sterile males that have a better flight ability than wild ones would improve their mating success, and increase the efficiency of the SIT [18]. Flight ability of moths can vary with age [13,19], and therefore, assessing the flight ability of *G. molesta* at different ages will allow the selection of the best time to release the sterile males in SIT programs.

In this study, the ovarian development, fecundity, and vitellogenin (precursor protein of egg yolk) content of *G.* *molesta* females of different age were assessed. An insect flight mill system was used to study the flight performance of *G. molesta* adult males and females of different ages. The cumulative flight distance, cumulative flight time, maximum flight distance, maximum flight duration, and average flight speed were the parameters assessed. The knowledge gained will assist project managers to make more informed decisions in SIT release programs involving the mass-produced males for release, and mass rearing programs involving the mass produced females and males to maintain the colonies.

## 2. Materials and Methods

### 2.1. Insects

The *G. molesta* moths were originally collected from peach orchards in Yangling (34°15′53′ N, 108°.3′42′ E), Shaanxi Province, China. The laboratory strain of *G. molesta* was reared on an artificial diet (Table S1) [20] in a climate-controlled incubator (GZC-500A model, Youke Company, Hefei, Anhui, China) that was maintained at 26 ± 1 °C, 70 ± 10% RH and a photoperiod of 15:9 (L:D) h for more than 30 generations. The adults of *G. molesta* were maintained in 5 L transparent glass flasks (9.5 cm in upper diameter, 17.6 cm in bottom diameter, 33.3 cm in height) (Renyuan Company, Cangzhou, Hebei, China) for mating and oviposition. The cotton (2.5 cm × 2.5 cm) (Hualu Company, Caoxian, Shandong, China) soaked with 3 mL of honey solution (10%) in a plastic Petri dish (5 cm in diameter, 1 cm in height) were put at the bottom of the flash to supply food supplement to the adults.

### 2.2. Vitellogenin Levels

Enzyme-linked immunosorbent assay (ELISA) was used to determine the levels of vitellogenin in female adults of different age (one to seven days old). Three female adults of each age (one to seven days old) were homogenized by an electric grinder on ice (1 g tissue was homogenized with 9 mL 0.9% saline), respectively. The homogenate was centrifuged (5804R model, Eppendorf AG, Hamburg, Germany) at 2300× *g* for 15 min at a temperature of 4 °C, and the supernatant was taken for ELISA [21]. The instructions of the ELISA kit (Shanghai FANKEL Industrial Co., Ltd., Shanghai, China) were followed. The experiment was replicated three times. The absorbance of each sample was measured at 450 nm using a microplate reader (Tecan Infinite M200, Männedorf, Zurich, Switzerland), and the level of vitellogenin in female adults of different age was calculated by a standard curve [22].

### 2.3. Ovary Development

One- to seven-day-old female adults were dissected in a glass Petri dish (diameter of 90 mm) (Renyuan Company, Cangzhou, Hebei, China) containing PBS (phosphate buffer saline) buffer (Shenggong Biological Engineering Co. Ltd., Shanghai, China) using a ZSA300T Stereomicroscope (Zhong Xian Company, Chongqing, China). A tip tweezer (Shenggong Biological Engineering Co. Ltd., Shanghai, China) was used to hold the thoraco-abdominal junction of the moth, and another tip tweezer was used to remove the cephalothorax. The tweezers were then used to open the abdominal epidermis along the thoracic-abdominal junction to reveal the abdominal cavity, and to pull out the ovary and remove other tissues around the ovary [23]. The ovaries were transferred to a glass slide with a drop of PBS buffer. The glass slide was placed under a Zeiss SteREO Discovery V20 stereomicroscope (Carl Zeiss AG, Oberkochen, Baden-Württemberg, Germany) for the photographing of the ovaries. Ten females of each age were dissected.

### 2.4. Daily Fecundity

Male and female pupae were placed in separate transparent plastic cups (240 mL, 7.3 and 5.2 cm in diameter at both ends, 8.5 cm in height) (Renyuan Company, Cangzhou, Hebei, China). Newly emerged male and female adults were paired at random in the cup (240 mL, 7.3 and 5.2 cm in diameter at both ends, 8.5 cm in height) (Renyuan Company, Cangzhou, Hebei, China) for mating and oviposition. A 10% honey solution was provided to the adults as a nutritional supplement. The inner wall of the cup was covered with wax paper (Tiancheng Company, Yangling, Shaanxi, China) for egg laying. The experiment was replicated with 30 pairs, with the oviposition cups changed daily and the fecundity (no. eggs oviposited per female) recorded daily for seven days.

### 2.5. Flight Ability

One to seven-day-old female and male adults were used to determine the flight performance of the moths of different age. A 24-way computer-linked flight mill (Jiaduo Industry and Trade Co., Ltd., Hebi, Henan, China) was used in the experiment following the method described previously [24]. The experiment with the flight mills was carried out at 26 °C and 70 % RH. Flight parameters (the cumulative flight distance, cumulative flight time, maximum flight distance, maximum flight duration, and average flight speed) of the female and male adults of different age were automatically recorded by the computer program of the flight mill system (Jiaduo Industry and Trade Co., Ltd., Hebi, Henan, China) in darkness for 12 h. All the moths were individually put in a plastic Petri dish (5 cm in diameter, 1 cm in height) with pieces of cotton (2.5 cm × 2.5 cm) (Hualu Company, Caoxian, Shandong, China) soaked with 3 mL of honey solution (10%) before the test. Thirty male and 30 female adults of each age were used in the experiment. The flight parameters were automatically recorded by the flight mill information system.

### 2.6. Statistical Analysis

The data of flight parameters that did not meet the assumptions of normality and homoscedasticity were analyzed by the nonparametric Kruskal–Wallis test [25]. Data on the daily fecundity and vitellogenin content, which met the assumptions of normality and homoscedasticity, were analyzed by one-way analysis of variance (ANOVA). Tukey’s honestly significant difference (HSD) test (*p* < 0.05) was used to analyze significant differences among the different ages. All the data were analyzed using the SPSS version 28.0 software program (IBM Corp., Armonk, NY, USA). The quadratic model in SPSS 28.0 software was used to do regression analysis for testing the effect of age on the cumulative flight distance, cumulative flight time, maximum flight distance, maximum flight duration of *G. molesta* female and the effect of age on the average flight speed of *G. molesta* male The logarithmic model in SPSS 28.0 software was used to conduct the regression analysis for the testing effect of age on the cumulative flight distance, cumulative flight time, maximum flight distance and maximum flight duration of the *G. molesta* male. The linear model was used to conduct a regression analysis for the testing effect of age on the average flight speed of the *G. molesta* female.

## 3. Results

### 3.1. Vitellogenin Levels

**Figure 1:**
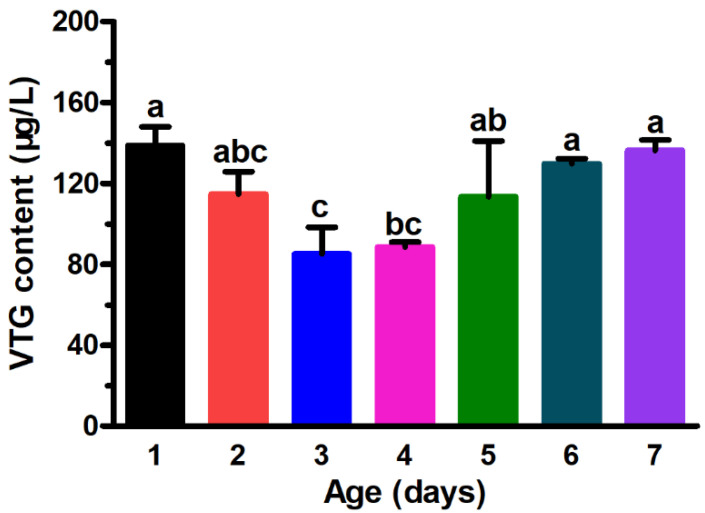
The vitellogenin (VTG) levels of *Grapholita molesta* females of different ages. The data are shown as the mean ± standard error. The different lower-case letters above the histogram bars indicate significant differences between VTG levels in females of different ages (ANOVA: HSD test, *p* < 0.05).

The vitellogenin levels of three-day-old females were significantly lower than that of one-, five-, six-, and seven-day-old females. One-day-old female adults had the highest vitellogenin content (138.77 μg/L), whilst the three-day-old female adults had the lowest vitellogenin level (85.48 μg/L) (Figure 1).

### 3.2. Ovary Development

**Figure 2:**
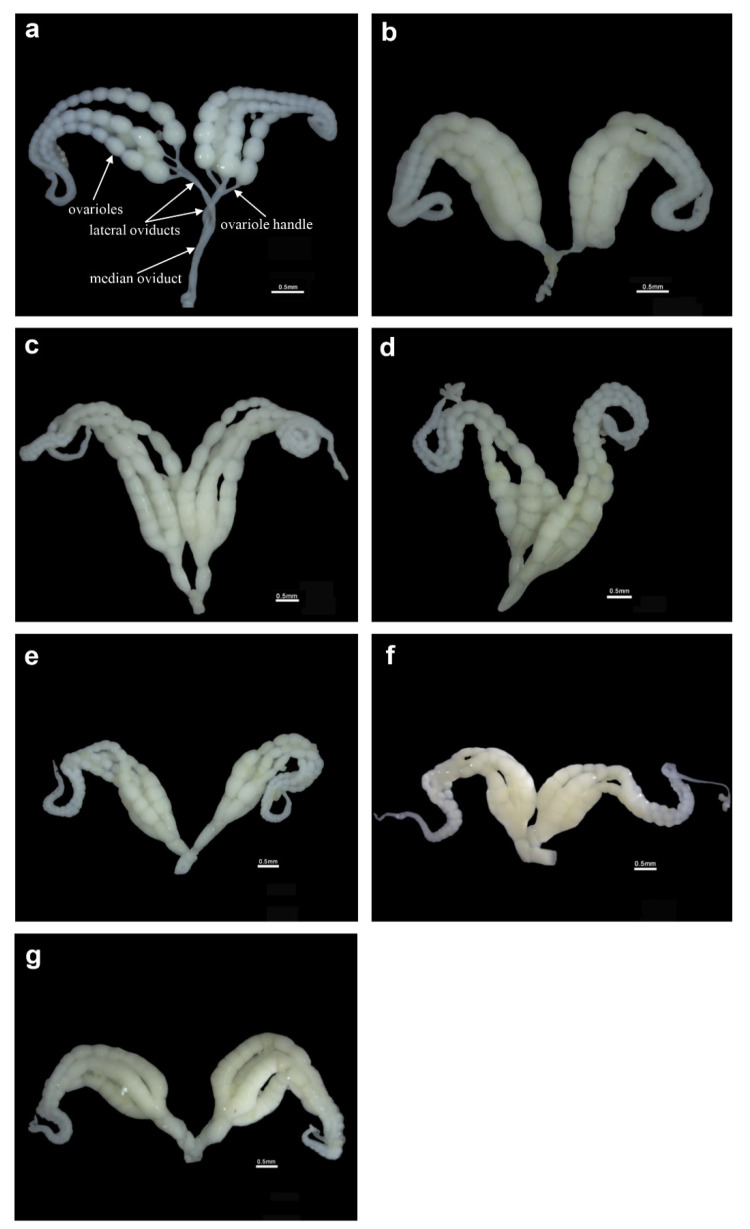
Ovarian development in *Grapholita molesta* females of different ages. (**a**) ovary of one-day-old female, (**b**) ovary of two-day-old female, (**c**) ovary of three-day-old, female, (**d**) ovary of four-day-old female, (**e**) ovary of five-day-old female, (**f**) ovary of six-day-old female, and (**g**) ovary of seven-day-old female. Bars = 0.5 mm. The structure of the ovary is marked with a arrow in Figure 2a.

*Grapholita molesta* females have a pair of ovaries, each consisting of four ovarioles. Figure 2 illustrates the development of the ovaries with increasing female age. The ovarioles of one-day-old moths were slender and the ovariole handle, lateral oviducts and median oviduct were all nearly transparent and there were no eggs inside. The eggs at the base of the ovariole were larger, and the growth area at the end of the ovariole had not yet formed an egg chamber (a). The ovarioles of two-day-old females were enlarged and thicker, and more compact as compared with those of one-day-old females; the eggs were closely arranged in the ovarioles without space between them. Additionally, the eggs in the lower part of the ovariole had matured and entered the ovariole handle (b). In three to seven-day-old females, the ovariole handle, the lateral oviducts, and the median oviduct had expanded, and the egg cells in the ovarioles were filled with the ovariole handle, lateral oviducts, and median oviduct (c to g).

### 3.3. Fecundity

**Figure 3:**
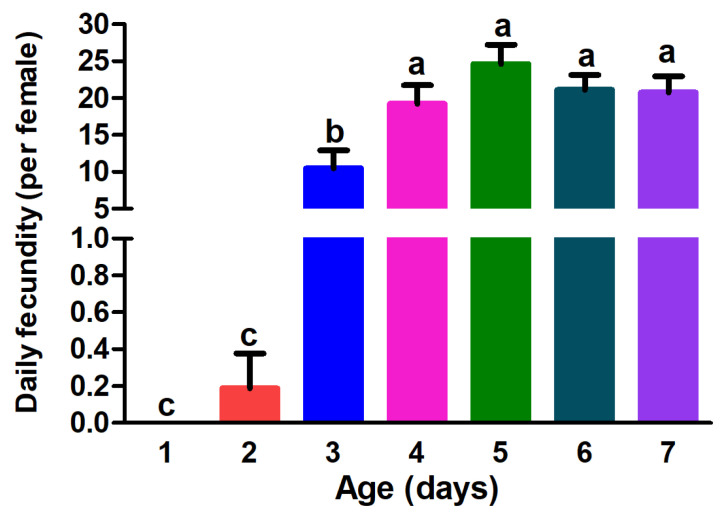
The fecundity of *Grapholita molesta* females of different ages. The data are shown as the mean ± standard error. The different lower-case letters indicate significant differences between the fecundity of females of different ages (ANOVA: HSD test, *p* < 0.05).

Four- to seven-day-old females oviposited significantly more eggs than one- to three-day-old females. The number of eggs laid by the one-day-old (0 egg) and two-day-old *G. molesta* (0.19 egg) was significantly lower than that of the three-day-old *G. molesta* (10.48 eggs). Five-day-old females oviposited the most eggs (24.6 eggs), whereas one-day-old females oviposited the least (Figure 3).

### 3.4. Flight Ability

**Figure 4:**
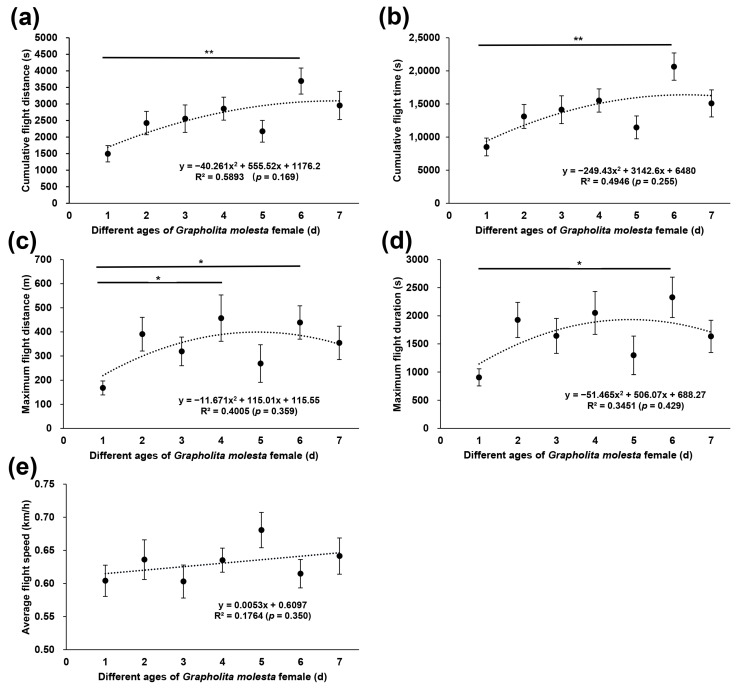
The cumulative flight distance (**a**), cumulative flight time (**b**), maximum flight distance (**c**), maximum flight duration (**d**), and average flight speed (**e**) of *Grapholita molesta* females of different age. The data are shown as the mean ± standard error. **, significant difference at *p* < 0.01; *, significant difference at *p* < 0.05.

**Figure 5:**
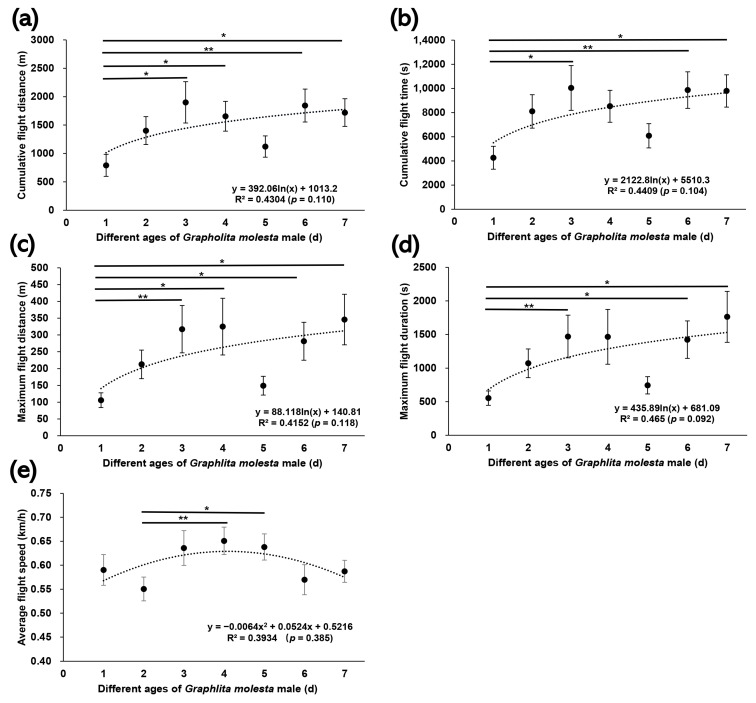
Cumulative flight distance (**a**)**,** cumulative flight time (**b**), maximum flight distance (**c**), maximum flight duration (**d**), and average flight speed (**e**) of male *Grapholita molesta* of different ages. The data are shown as the mean ± standard error. **, significant difference at *p* < 0.01; *, significant difference at *p* < 0.05.

The mean largest cumulative flight distance and longest cumulative flight time in the flight mill test were observed for six-day-old females (3691.91 m and 5.73 h, respectively) and for three-day-old males (1899.42 m and 2.79 h, respectively) (Figure 4 and Figure 5). The smallest cumulative flight distance and shortest cumulative flight time were observed for one-day-old female and male adults (1494.22 m, 2.36 h for females and 790.79 m, 1.18 h for males, respectively). Except for the average flight speed (Kruskal–Wallis H-test, *H* = 9.0, *p* = 0.17), the cumulative flight distance (*H* = 19.3, *p* < 0.01), cumulative flight time (*H* = 21.0, *p* < 0.01), maximum flight distance (*H* = 15.5, *p* < 0.05), and maximum flight duration (*H* = 14.8, *p* < 0.05) of *G. molesta* female adults were significantly different for each age. The cumulative flight distance (*H* = 12.8, *p* < 0.05), cumulative flight time (*H* = 13.6, *p* < 0.05), maximum flight distance (*H* = 13.0, *p* < 0.05), maximum flight duration (*H* = 13.0, *p* < 0.05), and average flight speed (*H* = 12.9, *p* < 0.05) of *G. molesta* male adults were significantly different for each age. The cumulative flight distance (*H* = −82.6, *p* < 0.01), cumulative flight time (*H* = −85.8, *p* < 0.01), maximum flight distance (*H* = −67.6, *p* < 0.05) and maximum flight duration (*H* = −67.2, *p* < 0.05) of six-day-old females were significantly higher than those of one-day-old females. The cumulative flight distance and maximum flight distance of one-day-old males was significantly lower than that of the three-day-old males (*H* = −62.7, *p* < 0.05 for cumulative flight distance, and *H* = −67.9, *p* < 0.01 for maximum flight distance), four-day-old males (*H* = −49.5, *p* < 0.05 for cumulative flight distance, and *H* = -51.1, *p* < 0.05 for maximum flight distance), six-day-old males (*H* = −62.4, *p* < 0.01 for cumulative flight distance, and *H* = −57.2, *p* < 0.05 for maximum flight distance), and seven-day-old males (*H* = −52.6, *p* < 0.05 for cumulative flight distance, and *H* = −59.1, *p* < 0.05 for maximum flight distance), respectively. The cumulative flight time and maximum flight duration of one-day-old males were significantly lower than those of the three-day-old males (*H* = -61.9, *p* < 0.05 for cumulative flight time, and *H* = −65.3, *p* < 0.01 for maximum flight duration), six-day-old males (*H* = −63.6, *p* < 0.01 for cumulative flight time, and *H* = -58.5, *p* < 0.05 for maximum flight duration), and seven-day-old males (*H* = −54.0, *p* < 0.05 for cumulative flight time, and *H* = −59.2, *p* < 0.05 for maximum flight duration), respectively. There was no significant correlation between the flight ability parameters and age (Figure 4 and Figure 5).

## 4. Discussion

For the SIT to be successful, the flight ability of the released sterile male insects should be as close as possible to that of the wild insect population. Flight ability is therefore one of the most important quality control parameters for sterile mass-reared males [26,27]. This is especially the case for sedentary moth species such as *G. molesta*, which are relatively poor fliers. Flight ability of sterile male insects is commonly measured using the two parameters of cumulative flight distance and cumulative flight time [28,29]. Often, the age of female adults that demonstrates the best flight ability is different from that of males. In this study, we found that six-day-old female adults and three-day-old male adults were the best fliers.

The age of male and female adults that had the lowest flight ability was the same, i.e., one-day-old adults. Similar to our results, the flight ability of both male and female *Pseudaletia unipuncta* (Lepidoptera: Noctuidae) was lowest when one day old [30]. The additional supplement such as the sterile water or honey solution is important for the flight capacity of the *G. molesta* moths [31]. These supplements can supply energy directly or promote the metabolizing of energy resources (lipids or carbohydrates) [31,32]. We provide the newly-emerged moth with supplement (10% honey solution) in this study. The one-day-old *G. molesta* moths could obtain more energy after feeding the supplement, and thus fly a longer distance later.

The flight ability of an insect species can be affected by a lot of factors, which include the age of the moth, flight fuels (carbohydrate, trehalose, or Glycogen) storage, level of hormones controlling flight fuel metabolism, tracheal system supplying oxygen to flight muscle mitochondria, water balance capacity during flight, and power output of flight muscles [32]. The relationship between age and the flight ability varies with species [33], but the flight ability of *Carposina sasakii* (Lepidoptera: Carposinidae) [34], *Ostrinia furnacalis* (Lepidoptera: Pyralidae) [35], *Homana magnanima* (Lepidoptera: Tortricidae) [36], *Rhynchophorus ferrugineus* (Coleoptera: Dryophthoridae) [37], and *Spodoptera exigua* (Lepidoptera: Noctuidae) [33] was not significantly different among adults at different ages of the respective species. However, both female and male adults of *Spodoptera litura* (Lepidoptera: Noctuidae) had the strongest flight ability when three days old [13]. Five-day-old female adults of *Microplitis mediator* (Hymenoptera: Braconidae) had the strongest flight capacity [38]. Further analyses are needed to detail the factors and mechanisms which affect the flight ability of the moth species at different ages. In the *Grapholita molesta* population, most individuals in a population have a limited flight ability and are qualified as short fliers, but a small portion of individuals have the ability to disperse between orchards [39,40]. For applying the SIT against moth species, we require that the immigration of wild moths into the treated areas be kept to a minimum, and that the sterile male can fly some distance to locate and mate the wild female moths [41,42]. Knowledge of the moth quality control parameters including the flight ability is important for the mass-rearing of *G. molesta* for SIT.

Other important quality control parameters with respect to the mass-rearing of insects for the SIT are the fecundity and ovarian development of female adults. The ovary is the organ where eggs develop in the female reproductive system, and the number of eggs (fecundity) can vary among adult females of different ages [43]. The fecundity of *Dryocosmus kuriphilus* (Hymenoptera: Cynipidae) [43] and *Thermophilus javanus* (Hymenoptera: Braconidae) [44] significantly changes with female adult age. The adult age that can affect the fecundity was found in some moth species. For example, the age of *Lymantria dispar* (L.) (Lepidoptera: Lymantriidae) females and males can affect the fecundity of the moth [45]. For *Micromelalopha troglodyta* (Graeser) (Lepidoptera: Notodontidae), eggs laid by females at the age of 1 to 3 days accounted for 68.4% of their total lifetime fecundity [46]. Our results demonstrate that four-day-old to seven-day-old *G. molesta* moths had the highest fecundity. Ovarian development is a highly dynamic process [47]. In this study, we found that the fecundity of one- to two-day-old *G. molesta* females was significantly lower than that of three to seven-day-old moths, and no eggs were oviposited by one- to two-day-old females. The ovaries had no eggs in the lateral oviducts and the middle oviduct when one to two days old. However, the mature eggs appeared in the lateral and middle oviducts of the ovaries three days post-emergence and the females can lay eggs on that day.

## 5. Conclusions

In conclusion, our study demonstrated that the flight performance and fecundity of *G. molesta* moths changed with age. Similarly, ovarian development was also different with age. The fecundity of *G. molesta* at different ages was closely related to the ovarian development. *Grapholita molesta* females began to lay eggs in large numbers from the third day after emergence. It is suggested that three-day-old males are the best option for releasing *G. molesta* for SIT. When mass-rearing and keep population of the moth in the factory, female moths older than three days can be used for egg laying.

## Data Availability

The data presented in this study are available on request from the corresponding author.

## Supplementary Materials

The following supporting information can be downloaded at: https://www.mdpi.com/article/10.3390/insects13090837/s1, Table S1: Artificial diet formula that was used in this study.
